# Camptocormia as a Phenotypic Variant of FSHD in the Elderly: Clinical, Genetic, and Imaging Features

**DOI:** 10.1111/ene.70332

**Published:** 2025-10-02

**Authors:** Eleonora Torchia, Patrick Vandeputte, Mauro Monforte, Charlène Gillet, Christophe Verny, Rafaelle Bernard, Giorgio Tasca, Marco Spinazzi

**Affiliations:** ^1^ Department of Neuroscience Università Cattolica del Sacro Cuore Rome Italy; ^2^ Neurology Department CHU d'Angers Angers France; ^3^ Neuromuscular Reference Center, Department of Neurology CHU d'Angers Angers France; ^4^ UOC di Neurologia Fondazione Policlinico Universitario A. Gemelli IRCCS Rome Italy; ^5^ Molecular Genetics Laboratory CHU La Timone Marseille France; ^6^ John Walton Muscular Dystrophy Research Centre, NIHR Newcastle Biomedical Research Centre Newcastle University and Newcastle Hospitals NHS Foundation Trusts Newcastle Upon Tyne UK; ^7^ MITOLAB INSERM U1083 Angers France

**Keywords:** aging, atypical phenotype, camptocormia, facioscapulohumeral muscular dystrophy, FSHD, muscle disorders, myopathy

## Abstract

**Background and Objectives:**

Camptocormia, a pathological forward flexion of the spine, is a relatively common but often unexplained postural abnormality. Facioscapulohumeral muscular dystrophy (FSHD), one of the most prevalent adult myopathies, is caused by a contraction of D4Z4 repeats on chromosome 4 and typically presents with facial, scapular, and lower limb weakness. However, atypical phenotypes are increasingly recognized. We investigated camptocormia as a presenting feature of FSHD in a large neuromuscular cohort.

**Methods:**

This cross‐sectional study assessed clinical, genetic, and muscle imaging features in patients with FSHD presenting with camptocormia and compared them to patients with typical FSHD.

**Results:**

Among 87 patients with genetically confirmed FSHD, 8 (9.1%) had camptocormia as the predominant and initial manifestation. FSHD also accounted for 47% (8/17) of all cases of camptocormia due to axial myopathy. Compared to classical FSHD, camptocormic patients exhibited later disease onset, moderately contracted D4Z4 repeats, and marked axial involvement, with predominant spinal extensor weakness and relatively preserved abdominal strength. Muscle MRI revealed more severe paravertebral involvement and milder serratus anterior involvement than typically observed in FSHD.

**Conclusions:**

Camptocormia represents a relatively frequent and distinct phenotypic variant of FSHD, particularly in older adults. Conversely, FSHD is a common cause of camptocormia due to axial myopathy. These findings expand the clinical spectrum of FSHD and underscore the importance of considering FSHD in the differential diagnosis of camptocormia, even in the absence of typical clinical signs of FSHD. Muscle imaging may assist in identifying FSHD‐associated camptocormia.

## 1 Introduction

Camptocormia—derived from the Greek camptos (bent) and cormos (trunk)—refers to a pathological forward flexion of the thoracic and lumbar spine that is fully reversible when lying down but worsens in a standing position. This term is also interchangeably used with bent spine. First described by Brodie in 1818 [1], the term gained prominence during World Wars I and II, when it was commonly used to describe psychogenic postural abnormalities caused by combat‐related stress. For decades, camptocormia was implicitly associated with conversion disorder. It was not until 1992 that the term was applied to cases involving myopathy of the paravertebral muscles [2]. Few years later, camptocormia was reported in eight patients with Parkinson's disease [3]. Today, camptocormia is recognized as a clinical sign that can result from a heterogeneous group of conditions leading to weakness of the paraspinal muscles [4]. The most common causes include movement disorders such as Parkinson's disease [5], neuromuscular and degenerative joint conditions, while psychogenic camptocormia has become exceedingly rare [6, 7]. Although degeneration of axial muscles is frequent in the natural history of several muscle diseases as part of a widespread generalized muscle involvement [8], more rarely specific myopathies can present with camptocormia due to a predominant or exclusive axial muscle involvement [9, 10]. Most cases of camptocormia caused by axial myopathy remain, however, a diagnostic dilemma [10]. Sporadic cases of camptocormia associated with facioscapulohumeral muscle dystrophy (FSHD) have been described [11, 12, 13, 14]. FSHD is one of the most prevalent genetic myopathies among the adult population. In its most frequent form, FSHD type 1 (FSHD1), it is associated with a shortening of a microsatellite repeat sequence at the D4Z4 locus in the subtelomeric region of chromosome 4 (4q35). Clinically, it typically manifests in young adults with a variable combination of facial, scapular, humeral, and lower limb muscles [15], although other muscles can also be involved by the disease, and atypical phenotypes are increasingly recognized [16]. However, the relative frequency of camptocormia as an atypical presentation in FSHD is unknown, and it is not clear whether camptocormic patients with FSHD present distinctive clinical and molecular features compared with typical patients with FSHD. To address this gap, this study investigated the frequency of camptocormia as the presenting sign of FSHD among patients followed in a large French neuromuscular center and defined the clinical, molecular, and muscle imaging signatures of this clinical phenotype compared to typical FSHD cases. We show that camptocormia is a relatively frequent presentation of FSHD and that FSHD represents a significant cause of axial myopathy in our cohort. Moreover, we defined the clinical, genetic, and muscle imaging features of FSHD‐related camptocormia, representing a defined phenotypic variant of FSHD in the elderly.

## 2 Materials and Methods

### 2.1 Study Population

We identified 87 adult patients affected by FSHD1 seen between June 2011 and February 2025 at the Neuromuscular Diseases Reference Center of the Angers University Hospital. Diagnosis of FSHD1 was based on the presence of a compatible clinical phenotype [15], with molecular confirmation of a D4Z4 locus with < 11 repeats [17]. Asymptomatic carriers of D4Z4 contractions were not included. Neuromuscular assessment was performed by a neurologist specialized in neuromuscular diseases with extensive experience in neurogenetic diseases. Motor function assessment included quantification of muscle strength by Medical Research Council (MRC) evaluation, 10‐m walking test, measurement of daily life activity limitation by ACTIVLIM [18], and assessment of the presence of typical FSHD signs, such as facial weakness, scapular winging, Beevor's sign [19], and hyperlordosis. Beevor's sign was considered present when observing an abnormal upward umbilicus movement upon truncal flexion [19]. Hyperlordosis was defined as a pathological increase in lumbar lordosis. MRC sum score was calculated as the sum of individual MRC scores from 17 muscles (adapted from [20]: 4 axial muscles, 5 upper limb muscles, 8 lower limb muscles). Typical FSHD presentation was defined by onset with weakness, often asymmetric, at the level of facial, scapular girdle, humeral, or tibialis anterior muscles, hyperlordosis, and abdominal muscle weakness in a variable combination [15, 21]. Patients with insufficient clinical data or severe coexisting comorbidities interfering with the functional motor capacity were excluded. Camptocormia was defined by an abnormal thoraco‐lumbar kyphosis in the upright position fully reversible in the supine position. Laboratory tests were performed in patients with unexplained camptocormia to identify alternative metabolic and autoimmune etiologies: blood calcium levels, PTH, TSH, lactate, myositis antibody profile, acid maltase activity to rule out Pompe disease, anti‐acetylcholine receptor (AChR), and muscle‐specific kinase (MuSK) antibody assays. Camptocormia was attributed to axial myopathy based on the following criteria: (1) exclusion of alternative conditions explaining camptocormia, such as movement disorders, motor neuron disease, orthopedic causes, neuromuscular junction defects, psychogenic, iatrogenic, or metabolic diseases; extrapyramidal involvement was excluded by medical history, careful neurological examination, and follow‐up; and (2) the presence of muscle weakness of spine extensor muscles associated with significant fatty replacement of paravertebral muscles on muscle imaging. Diagnosis of FSHD‐related camptocormia was based on the following criteria: (a) a positive molecular test for FSHD; (b) coexistence of the previous two criteria fulfilling the definition of camptocormia of muscular origin; (c) camptocormia was the main presenting symptom; (d) exclusion of myositis or alternative myopathy diagnosis based on the clinical, radiological, genetic, and biochemical evaluations and follow‐up. FSHD clinical scores and clinical categories were assessed as described [22].

### 2.2 Standard Protocol Approvals, Registrations, and Patient Consents

This study was conducted in accordance with the declaration of Helsinki. Patients were informed that the results of their medical exam were susceptible to being used for research purposes and could exercise their right to disagree. As such, and according to French law, no informed consent was required. The retrospective collection of the data was authorized by the local ethic committee under reference 2025‐005.

### 2.3 Muscle Imaging

Muscle imaging studies were performed through MRI or CT scans. MRI studies were performed according to standard protocols [23, 24] to cover the upper and lower body, and T1‐weighted sequences were analyzed. Whole‐body CT scans were acquired with a slice thickness of 3.0 mm and contiguous axial sections. All images were assessed to evaluate intramuscular fatty replacement and hypotrophy. A total of 51 muscles were independently analyzed on both sides for each scan by a neurologist with expertise in muscle imaging. The severity of muscle involvement was graded using a semiquantitative scoring system (T1‐score), applying a 4‐point scale for upper body muscles (cranial, neck, scapular girdle, and dorsal trunk) and a 5‐point scale for lower body muscles (pelvic girdle, lower limbs, and lumbar trunk) as previously described [25, 26]. Higher scores indicated greater fatty replacement and/or atrophy. To identify distinctive radiological features of patients with FSHD with camptocormia, we compared their imaging findings with previously published data from a large independent cohort of patients with FSHD [27]. A group of patients matched for whole body fatty replacement (global T1‐score, computed as the sum of the individual T1‐scores of all assessed muscles on both sides.) was identified in the published dataset and used for the analysis.

### 2.4 Statistical Analysis

For categorical variables, statistical significance of proportions comparison was calculated with the exact Fisher test. For continuous variables, differences between the two groups of patients were analyzed with either a Student's *t*‐test with Welch correction for normally distributed variables or a Mann–Whitney test for non‐normally distributed variables. Normality of the distribution was assessed using the Shapiro–Wilk test. Correction for multiple comparisons was performed with Benjamini–Hochberg post hoc analysis. Significance was considered for *p* values < 0.05. All statistical tests were computed with the Prism v8.3.0 software (GraphPad, San Diego, CA).

## 3 Results

### 3.1 FSHD Is a Frequent Cause of Late‐Onset Camptocormia Related to Axial Myopathy

**FIGURE 1:**
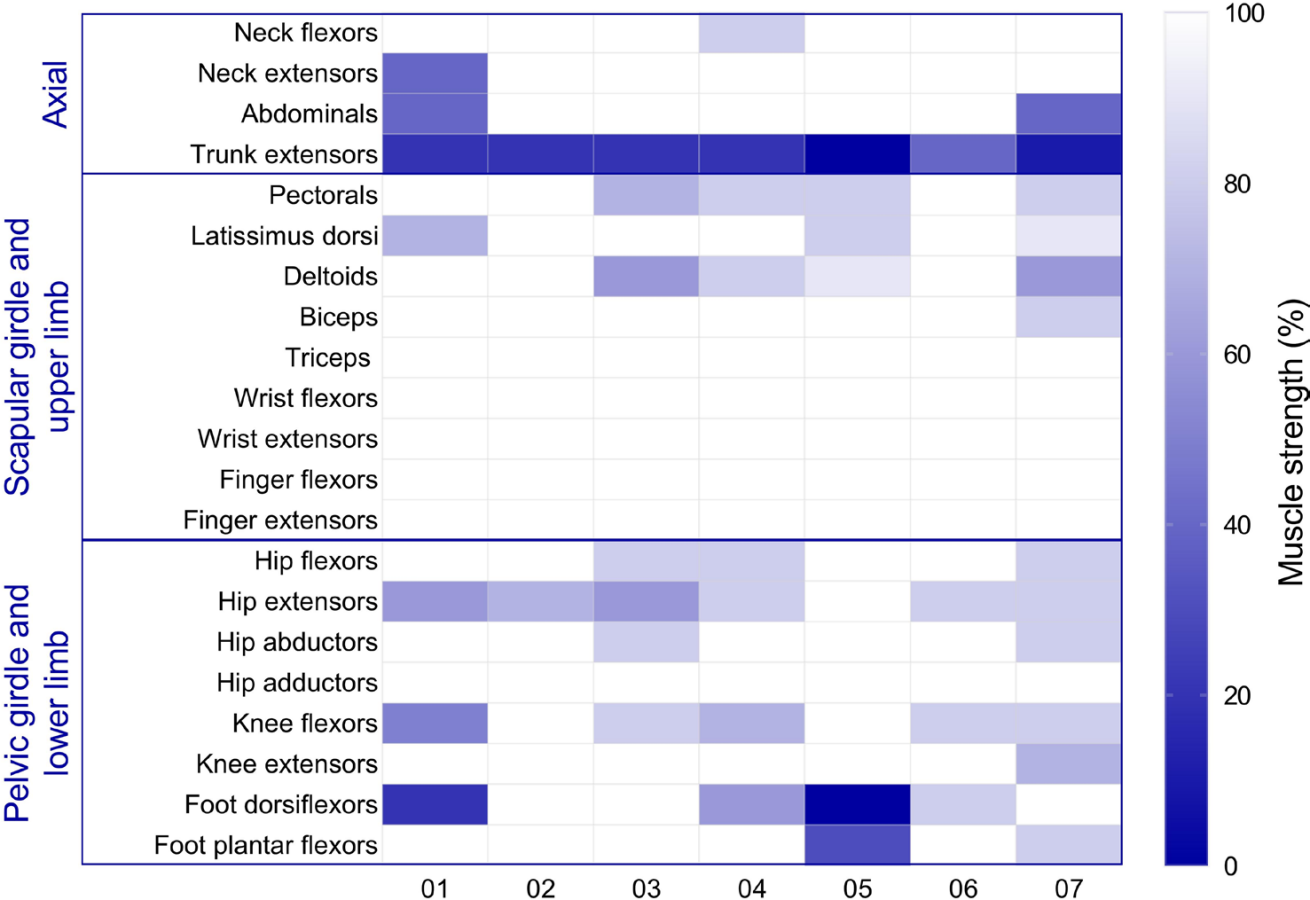
Pattern of muscle weakness involvement in camptocormic patients with FSHD. The heat map represents color‐coded muscle strength values evaluated through the Medical Research Council scale ranging from normal (100%, white, corresponding to MRC 5/5) to absence of clinically detectable muscle contraction (0%, dark blue, corresponding to MRC 0/5) in camptocormic patients. Individual values for each camptocormic patient are shown. The color legend is displayed on the right *y* axis.

Between 2011 and 2025, 87 individual adult patients with genetically confirmed FSHD1 were examined at the Neuromuscular Diseases Reference Center of the Angers University Hospital. Ten patients were excluded from the study due to insufficient available clinical data. Four additional patients were excluded due to coexisting comorbidities interfering with the functional motor capacity. Among the remaining 73 FSHD patients, 65 patients presented a typical phenotype, and 8 patients, representing 9.1% of 87 total patients with FSHD, presented camptocormia as the first and predominant manifestation of the disease (Figure S1). All patients with FSHD‐related camptocormia were investigated to rule out an alternative diagnosis, including movement disorders and neuromuscular disorders, and no alternative explanation for camptocormia was identified at the time of first evaluation or at follow‐up. Results of the electromyography (EMG) were available for 6/8 cases: 4 cases had myopathic EMG changes, one case had signs of L5‐S1 radiculopathy in the left leg, and in one case it was normal. All patients complained of impaired walking ability and progressive bending forward of the trunk during gait. They adopted compensatory strategies to keep the standing position or used walking aids: two patients walked holding their hands on their back, one patient holding their hands on the root of the thighs, one using a small backpack to improve his balance, one used two crutches for distances exceeding 200 m, and one walked only a few meters by leaning on nearby supports. All patients with FSHD‐camptocormia had been addressed to a rehabilitation specialist. A corset was proposed to most patients, but only a few found it comfortable enough to use it regularly. Two used a walker. They all presented abnormal thoraco‐lumbar kyphosis in the upright position, which was fully reversible in the supine position, and pelvis retroversion. We performed a detailed muscle strength assessment in 7/8 camptocormic patients, showing severe axial involvement with trunk extension weakness (Figure 1). Other muscles, typically affected in FSHD, such as pectoral, knee flexors, tibialis anterior, and abdominal muscles, were variably and inconsistently affected in FSHD camptocormia (Figure 1). Consequently, FSHD clinical scores varied between 0 and 8 (Table S1). Since all patients presented camptocormia, previously defined as an uncommon feature in the comprehensive clinical evaluation form of FSHD^22^, all patients were classified in category D, corresponding to atypical FSHD phenotypes. Five patients were classified as category D1 and three patients, lacking either winged scapula or facial involvement, as category D2 (Table S1).

To evaluate the contribution of FSHD to neuromuscular camptocormia, we retrospectively reviewed medical records from the Angers Neuromuscular Center and identified 17 patients diagnosed with axial myopathy presenting as camptocormia between 2011 and 2025. Among the 15 patients who underwent molecular testing for FSHD, 8 (53%) showed a D4Z4 contraction consistent with FSHD. Overall, FSHD accounted for 47% (8/17) of myopathic camptocormia cases in our cohort.

These data indicate that camptocormia is a relatively common atypical FSHD phenotype, and that FSHD represents a significant and possibly underrecognized cause of late‐onset camptocormia.

### 3.2 Distinctive Clinical and Genetic Features of Patients With FSHD Presenting With Camptocormia

To further investigate whether FSHD‐camptocormia shows distinct phenotypic and genetic signatures compared to typical patients with FSHD, we compared the clinical and molecular features of these two groups. Functional capacity, evaluated by walking speed as well as by the measure of daily life activity limitations (ACTIVLIM scores), was similar in the two groups, suggesting a comparable disease burden. Plasma creatine phosphokinase (CPK) levels and positive family history for FSHD were not different in the two groups. However, disease onset appeared much later in camptocormic patients with FSHD compared to typical patients with FSHD (59.6 ± 13.1 vs. 24.2 ± 17.5; *p* = 0.000006; Table 1). At the molecular level, camptocormic patients with FSHD had a significantly higher number of D4Z4 repeats compared to typical FSHD patients (8.1 ± 0.8 vs. 5.9 ± 2.1, *p* = 0.00001; Table 1). Camptocormic patients also less frequently showed facial involvement (50% vs. 92.3%; *p* = 0.006) and scapular winging compared to typical FSHD (12.5% vs. 67.7%, *p* = 0.004; Table 1). Beevor's sign, a sign of abdominal muscle weakness frequently found in typical patients with FSHD, was not detected in our camptocormic FSHD cohort (0% vs. 50.8%; *p* = 0.013; Table 1). Similarly, no camptocormic FSHD patient showed hyperlordosis (0% vs. 50.8% in typical FSHD, *p* = 0.007; Table 1).

**TABLE 1:** Clinical and genetic features of camptocormic FSHD versus typical FSHD patients.

Characteristics	Camptocormic FSHD (*n* = 8)	Typical FSHD (*n* = 65)	*p* (Significance)
Demographic and genetic features			
Age at onset (years)	59.6 ± 13.1	24.2 ± 17.5	0.000006 (****)
Male sex	5/8 (62.5)	33/65 (50.8)	0.712 (ns)
Family history of FSHD	5/8 (62.5)	36/65 (55.4)	1.0 (ns)
Number of D4Z4 repeat units	8.1 ± 0.8	5.9 ± 2.1	0.00001 (****)
Clinical features			
Ambulant	8/8 (100)	51/65 (78.5)	0.339 (ns)
10MWT (seconds)	11.9 ± 4.5	11.5 ± 10.6	0.838 (ns)
ACTIVLIM score	23.4 ± 3.2	21.3 ± 8.0	0.247 (ns)
CPK blood concentration (UI/L)	217.0 ± 73.7	247.8 ± 188.2	0.430 (ns)
Facial involvement	4/8 (50)	60/65 (92.3)	0.006 (**)
Scapular winging	1/8 (12.5)	44/65 (67.7)	0.004 (***)
Beevor's sign	0/7 (0)	24/46 (52.2)	0.013 (*)
Hyperlordosis	0/8 (0)	32/63 (50.8)	0.007 (**)

*Note:* Categorical variables are presented as the number of cases, with proportions of total in percent between parentheses, whereas continuous variables are presented as means ± standard deviation. For some patients, few data were not available. For categorical variables, statistical significance of proportions comparison was calculated with the exact Fisher test. For continuous variables, differences between means of the two groups of patients were analyzed with either a Student's *t*‐test with Welch correction for normally distributed variables or a Mann–Whitney test for non‐normally distributed variables. 10MWT, 10 m walking test; CPK, creatine phosphokinase blood concentration. n.s, not significant.

* *p* value < 0.05.

** *p* value < 0.01.

*** *p* value < 0.005.

**** *p* value < 0.0001.

**FIGURE 2:**
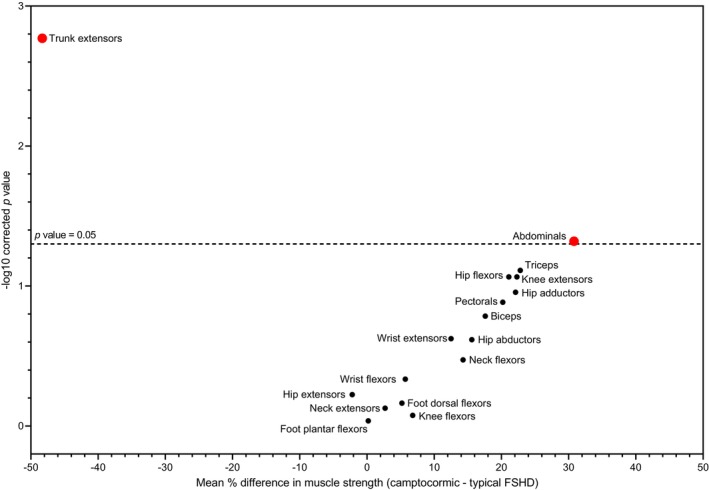
Differences in muscle weakness distribution in camptocormic FSHD versus typical patients with FSHD. The volcano plot shows the mean difference in muscle strength of bent spine FSHD (*n* = 7) versus typical patients with FSHD (*n* = 65). Statistical significance was calculated with a Mann–Whitney test with Benjamini–Hochberg post hoc analysis for correction of multiple comparisons. The *y* axis indicates the statistical significance (log_10_ of the *p* value with *p* < 0.05 considered significant). The *x* axis indicates the mean difference in muscle strength between the two groups (in %). Muscles weaker in the camptocormic group compared to typical FSHD are shifted to the left, while stronger muscles are shifted to the right side of the graph. Muscles differentially involved in the two groups are indicated in red above the dashed line. Compared to typical FSHD, camptocormic patients with FSHD have more severe trunk extensor muscle weakness and stronger abdominal muscles.

We next compared muscle strength between typical FSHD patients and those with FSHD‐related camptocormia to assess differences in the pattern of muscle involvement. Camptocormic patients exhibited significantly greater weakness of trunk extensor muscles (*p* = 0.0017) and less pronounced weakness of abdominal muscles (*p* = 0.047) (Figure 2). No statistically significant differences were observed in limb muscle strength.

Taken together, these findings indicate that FSHD patients with camptocormia display distinct clinical and molecular characteristics compared to typical cases, including later disease onset, longer D4Z4 repeats, more severe trunk extensor weakness, and less frequent involvement of facial, abdominal, and scapular muscles, as well as a lower prevalence of lumbar hyperlordosis.

### 3.3 Muscle Imaging Features in FSHD‐Related Camptocormia

**FIGURE 3:**
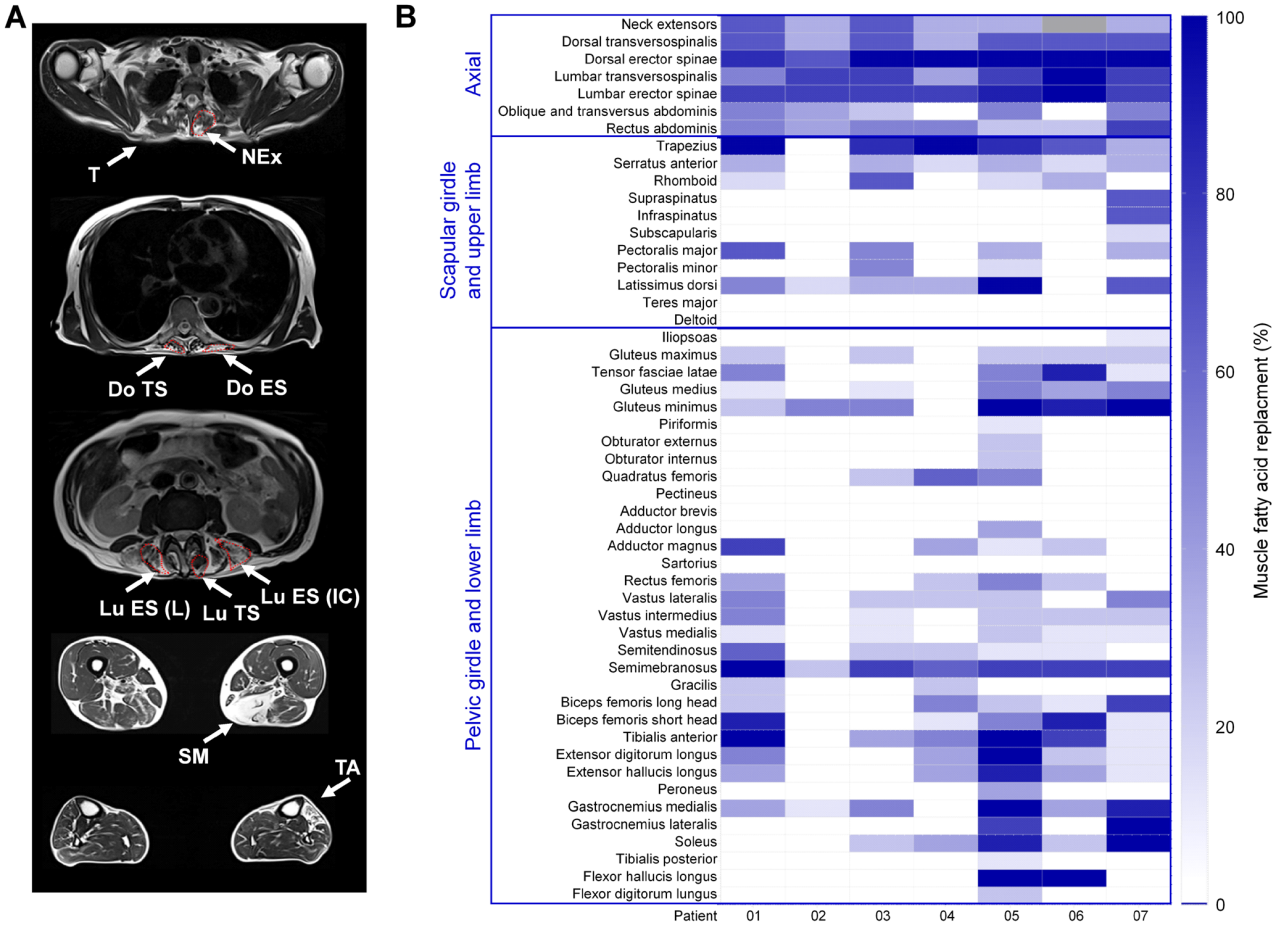
Muscle imaging pattern in camptocormic patients with FSHD. (A) Representative whole body muscle MRI showing the muscles typically involved in FSHD‐camptocormia: Trapezius (T), neck extensors (Nex), dorsal transversospinalis (Do TS), dorsal erector spinae (Do ES), lumbar transversospinalis (Lu TS), longissimus muscle of lumbar erector spinae (Lu ES‐L) and iliocostalis (Lu‐ES IC), semimembranosus (SM), and tibialis anterior (TA). Involvement of limb muscles is often asymmetrical. (B) Heat map representing color‐coded muscle fatty replacement of each muscle ranging from 0% (white, corresponds to absence of muscle fatty replacement) to 100% (dark blue, corresponding to complete fatty replacement) in FSHD‐camptocormia (*n* = 7). The color legend is displayed on the right hand side. Severe involvement of axial muscles and in particular of dorsal and lumbar erector spine muscles is evident. Neck extensor muscles could not be visualized in a single patient due to insufficient acquisition coverage (gray box).

Whole body muscle imaging was available for seven patients with FSHD with bent spine. A total of 714 muscles were assessed. Significant degeneration of paraspinal muscle was evident in all patients along the whole length of the spine, from cervical to lumbar, including neck extensor muscles. Overall, the erector spinae muscles were more affected than transversospinalis muscles (Figure 3A,B). Other muscles frequently affected in FSHD, including abdominal, trapezius, gluteus minimus, semimembranosus, and tibialis anterior, were frequently but variably affected (Figure 3A,B; Figure S2A). In many cases, the radiological involvement of these muscles was subclinical. Moreover, it varied significantly among patients in a broad spectrum of severity, ranging from severe (Figure S2A) to almost absent in a single patient, showing an almost exclusive axial involvement (Figure S2B).

**FIGURE 4:**
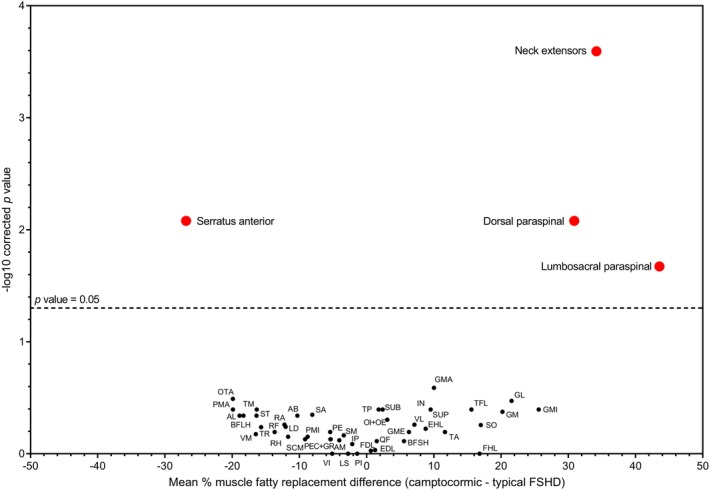
Comparison of muscle imaging involvement in camptocormic FSHD versus typical patients with FSHD. The volcano plot shows the mean difference in muscle fatty replacement scores of 51 muscles in bent spine FSHD (*n* = 7) versus typical patients with FSHD (*n* = 51). Statistical significance was calculated with a Mann–Whitney test with Benjamini–Hochberg post hoc correction. The *y* axis indicates the statistical significance (log_10_ of the *p* value with *p* < 0.05 considered significant). The *x* axis indicates the mean difference in T1‐score between the two groups. Muscles differentially involved in the two groups are indicated in red above the dashed line. Compared to typical FSHD, camptocormic patients with FSHD have more severe fatty degeneration of spine extensor muscles along the whole spine but more preserved serratus anterior muscles. AB, adductor brevis; AL, adductor longus; AM, adductor magnus; BFLH, biceps femoris long head; BFSH, biceps femoris short head; EDL, extensor digitorum longus; EHL, extensor hallucis longus; FDL, flexor digitorum longus; FHL, flexor hallucis longus; GL, gastrocnemius lateralis; GM, gastrocnemius medialis; GMA, gluteus maximus; GME, gluteus medius; GMI, gluteus minimus; GR, gracilis; IN, infraspinatus; IP, iliopsoas; LD, latissimus dorsi; LS, levator scapulae; OE, obturator externus; OTA, obliquus and transversus abdominis; PEC, pectineus; PE, peroneal; PI, piriformis; PMA, pectoralis major; PMI, pectoralis minor; QF, quadratus femoris; RA, rectus abdominis; RF, rectus femoris; RH, rhomboid; SA, sartorius; SCM, sternocleidomastoid; SM, semimembranosus; SO, soleus; ST, semitendinosus; SUB, subscapularis; SUP, supraspinatus; TA, tibialis anterior; TFL, tensor fasciae latae; TM, teres major; TP, tibialis posterior; TR, trapezius.

To investigate whether FSHD‐related camptocormia was characterized by any specific radiological pattern of muscle degeneration, we compared muscle imaging phenotypes with a previously published cohort of 181 patients with FSHD [27], from which we selected 51 typical patients with FSHD with global whole body muscle fatty replacement similar to the camptocormic group (global T1 score: 41.54 ± 11.6 in typical FSHD vs. 42.9 ± 19.3 in bent spine; *p* = ns). The bent spine group showed significantly higher muscle fatty replacement in thoracic paraspinal (*p* = 0.0083), lumbosacral paraspinal (*p* = 0.0021), and neck extensor muscles (*p* = 0.00025) (Figure 4). Conversely, fatty replacement was lower at the level of serratus anterior muscles in FSHD‐camptocormia compared to typical FSHD (*p* = 0.0083) (Figure 4). Altogether, these results indicate that patients with FSHD with camptocormia were more severely affected in paravertebral muscles with relative sparing of the serratus anterior muscle compared to typical FSHD, with no significant difference in the radiological involvement of the other muscles.

## 4 Discussion

The description of FSHD‐related camptocormia (bent spine) has been limited to sporadic case reports or small cohort studies, and comprehensive analyses of its clinical and genetic features are lacking. It has remained unclear whether these patients exhibit distinct clinical or molecular traits compared to typical FSHD cases, or if they represent an extreme of the phenotypic spectrum.

**FIGURE 5:**
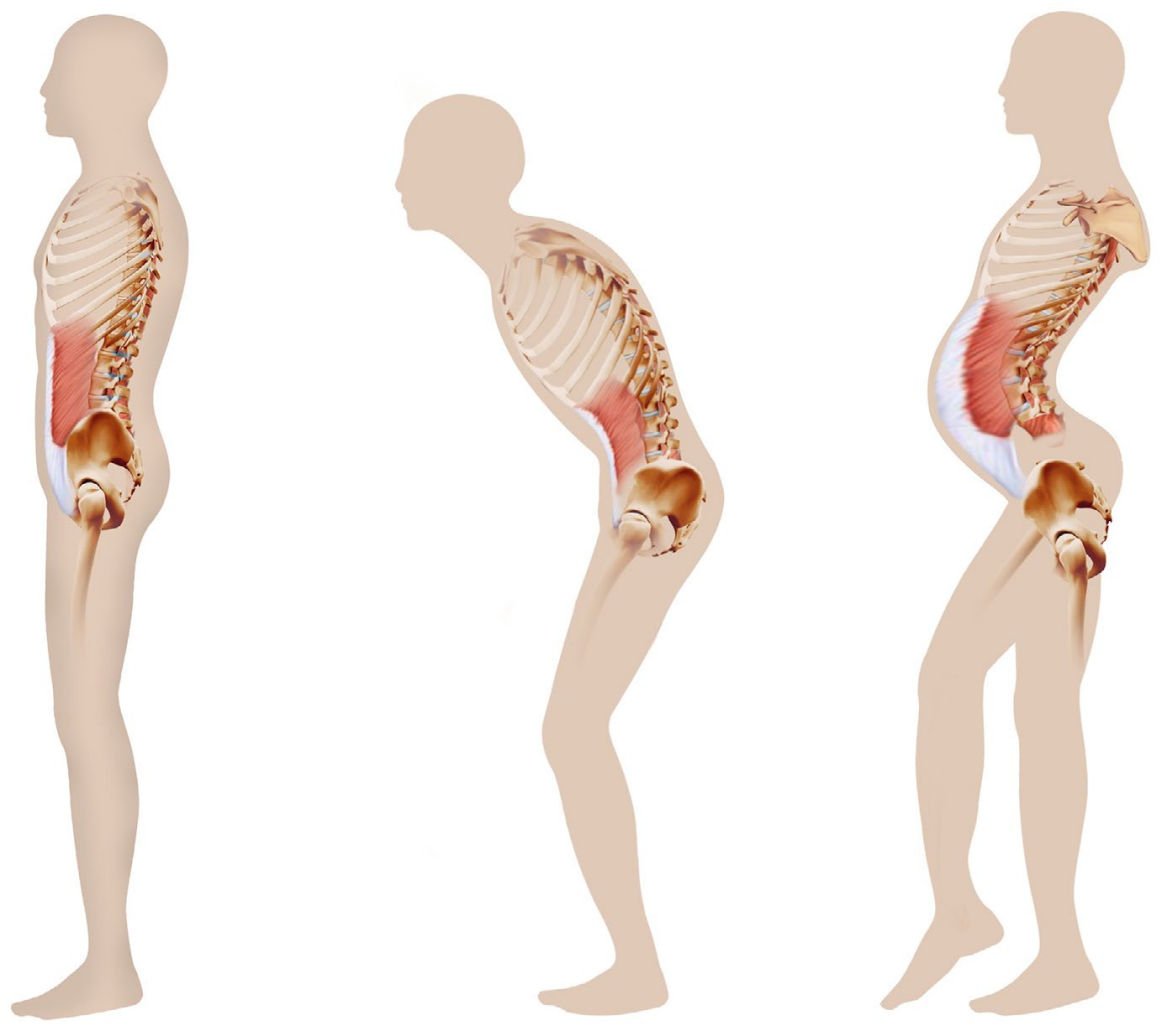
Distinct clinical phenotype and underlying mechanisms of FSHD camptocormia. In camptocormic patients with FSHD (middle), bent spine is caused by spine extensor muscle weakness, leading to dorso‐lumbar spine kyphosis, with inversion of the physiological lumbar spine lordosis present in normal individuals (left). Camptocormia is associated with compensatory backward tilt of the pelvis and hip flexion (middle). Patients with camptocormia commonly walk holding their arms behind their back or over their groin to improve postural stability of the trunk. In more severe cases, the use of a walker is required. Compared to typical FSHD (right), abdominal muscle strength is better preserved, contributing to these postural abnormalities. In typical FSHD, abdominal muscle weakness is more severe, leading to the presence of abdominal forward protrusion and positive Beevor's sign. In contrast with camptocormic FSHD, in typical FSHD (right), lumbar lordosis is accentuated and associated with anterior tilt of the pelvis. Other typical features of FSHD, such as facial involvement, winging scapulae, and limb muscle weakness, including foot drop, are less common in FSHD camptocormia and can be absent.

In this study, we systematically characterized a large cohort of FSHD patients with camptocormia and compared them to deeply phenotyped typical FSHD patients matched for disease burden. We demonstrate that camptocormic FSHD patients display significant clinical, molecular, and radiological differences from typical cases. Camptocormia occurred exclusively in elderly individuals, with a later disease onset and longer D4Z4 repeat fragments. Severe weakness consistently involved the spinal extensor muscles, while other commonly affected regions in FSHD—such as facial, scapular, and abdominal muscles—were less frequently involved (Table 1; Figures 1 and 5). This observation has important clinical implications, as the absence of typical FSHD signs may delay or obscure the diagnosis. Compared to normal subjects (Figure 5, left), camptocormic FSHD patients (Figure 5, middle) exhibit postural abnormalities distinct from those seen in typical FSHD cases (Figure 5, right). While typical FSHD is characterized by lumbar hyperlordosis and anterior pelvic tilt due to pelvic extensor weakness [28] (Figure 5, right) camptocormic patients display reversed spinal curvature, with lumbar kyphosis and posterior pelvic tilt (Figure 5, middle). Notably, dorsal kyphosis is reversible in the supine position. In our cohort, Beevor's sign—commonly observed in typical FSHD and indicative of abdominal muscle weakness [19]—was consistently absent, and abdominal strength was significantly better preserved in camptocormic patients. We propose that a marked imbalance between weakened spinal extensor muscles and relatively spared abdominal muscles underlies the development of camptocormia and its associated postural abnormalities (Figure 5, middle).

This study also provides a detailed muscle imaging characterization of FSHD‐related camptocormia. All patients showed severe fatty replacement of paravertebral muscles along the entire spine, particularly the spinal erector group, correlating with marked trunk extensor weakness. In contrast, other muscles commonly affected in FSHD [27, 29]—such as the trapezius, semimembranosus, gastrocnemius, and tibialis anterior—were less severely involved. One patient at the extreme of this spectrum exhibited isolated paravertebral muscle involvement with spared trapezius, despite a positive family history. These findings align with prior imaging studies [30], but our analysis additionally revealed significant involvement of neck extensor muscles—a feature not previously assessed. This supports the notion that head drop and camptocormia may share a common pathophysiological spectrum [13].

To identify distinctive radiological features of FSHD‐related camptocormia, we compared patterns of muscle fatty replacement between camptocormic and typical FSHD patients matched for overall disease burden. Although severe paravertebral muscle involvement has been reported in typical FSHD [31], our findings show that camptocormic patients exhibit markedly greater fatty degeneration of spinal erector muscles along the entire spine. In contrast, the serratus anterior was relatively spared, offering a structural basis for the predominant trunk extensor weakness and the less frequent occurrence of scapular winging in this subgroup.

The disproportionate involvement of spinal erector muscles in bent spine FSHD patients suggests a marked, though not exclusive, susceptibility of these muscles to degeneration. The mechanisms driving this selective vulnerability remain unknown. Notably, D4Z4 arrays of 8–10 repeats are relatively common in the general population; yet, only a minority of carriers develop muscle weakness [32] indicating that additional modifiers influence disease penetrance and expressivity. We hypothesize that paravertebral muscle degeneration may occur with incomplete penetrance, potentially triggered by aging and other, yet unidentified, genetic, epigenetic, or environmental factors.

The underlying cause of axial myopathy presenting with camptocormia often remains undetermined [10]. In our cohort, FSHD1 emerged as the most frequent diagnosis among patients with axial myopathy manifesting as camptocormia. This finding is consistent with a previous study of 35 patients with camptocormia and axial myopathy, where FSHD was identified as the single most common cause among cases with a confirmed diagnosis [9].

While a few case reports have described FSHD as a potential cause of [11, 12, 13, 14], the frequency of this phenotype among patients with a molecular diagnosis of FSHD has remained poorly characterized. One study identified only 2 cases of camptocormia among 139 FSHD patients [33], while another reported 3 cases with prominent axial involvement, including bent spine, among 244 carriers of borderline D4Z4 alleles (9–10 repeats), both symptomatic and asymptomatic [32]. A similar population study involving 422 individuals with 7–8 D4Z4 repeats found atypical signs in 72 cases [16], though detailed clinical and radiological data were lacking.

Comorbidities—either sporadic or genetic—can occasionally account for atypical FSHD presentations [34]. In our study, we excluded four patients with interfering comorbidities. In particular, none of the included patients showed clinical signs of extrapyramidal or other central nervous system signs, myotonia, early cataracts, ophthalmoplegia, severe respiratory insufficiency, and unusually high CPK levels (> 1000 UI/L) at presentation or during follow‐up. Although we did not perform comprehensive genetic testing, no clinical, biological, or family history data suggested the presence of coexisting genetic disorders that might explain camptocormia independently of FSHD. However, the potential contribution of additional genetic modifiers influencing phenotypic penetrance cannot be excluded and warrants further investigation in larger cohorts through whole‐exome or whole‐genome sequencing.

Notably, this atypical phenotype accounted for nearly 10% of our overall FSHD cohort. The reasons for this relatively high prevalence compared to previous studies remain unclear. Potential explanations include differences in methodology, population characteristics, or referral bias. Additionally, FSHD‐related camptocormia may be underdiagnosed, as bent spine is often misattributed to aging or orthopedic conditions—particularly in the absence of limb or facial weakness or a positive family history. This misattribution may delay referral to neuromuscular centers and hinder appropriate diagnosis. In this context, muscle imaging is a valuable tool for detecting selective degeneration of axial and other FSHD‐hallmark muscles [29], thereby informing further diagnostic evaluation.

In our experience, patients with camptocormia due to FSHD or other myopathies often benefit from assistive devices such as walkers or orthopedic corsets. However, corsets may be uncomfortable for some patients and impractical for daily use. We therefore recommend referral to a rehabilitation specialist to develop a personalized intervention plan aimed at improving gait impairment associated with camptocormia.

This study broadens the clinical spectrum of FSHD. Patient selection in clinical trials is often based primarily on genetic diagnosis, with limited attention to the considerable clinical heterogeneity that may influence disease progression. Individuals with late onset or atypical features—such as camptocormia—may follow a distinct trajectory of functional decline compared to those with classical presentations. Moreover, standard outcome measures may not adequately capture their specific functional impairments. Accounting for phenotypic variability in clinical trials design is therefore essential to ensure meaningful and interpretable results.

In conclusion, this study defines the clinical, genetic, and radiological features of a distinct phenotypic variant of FSHD presenting as bent spine in the elderly. These findings highlight the need to systematically consider FSHD in the differential diagnosis of late‐onset camptocormia of muscular origin. Timely recognition is crucial for appropriate management and genetic counseling.

## Author Contributions

**Eleonora Torchia:** data curation, formal analysis, investigation, methodology, validation, writing – review and editing. **Patrick Vandeputte:** data curation, formal analysis, investigation, methodology, validation, writing – review and editing. **Mauro Monforte:** data curation, formal analysis, methodology, validation, writing – review and editing. **Charlène Gillet:** data curation, investigation, methodology, writing – review and editing. **Christophe Verny:** investigation, writing – review and editing. **Rafaelle Bernard:** data curation, investigation, methodology, writing – review and editing. **Giorgio Tasca:** data curation; formal analysis; investigation; methodology; supervision; writing – review and editing. **Marco Spinazzi:** conceptualization; data curation; formal analysis; investigation; methodology; project administration; resources; supervision; visualization; writing – original draft preparation.

## Conflicts of Interest

The authors declare no conflicts of interest.

## Supporting information


**Figure S1:** Flowchart of the study.

**Figure S2:** Spectrum of muscle imaging abnormalities in FSHD camptocormia.

**Table S1:** FSHD clinical scores and clinical categories of FSHD camptocormic patients.

## Data Availability

The data that support the findings of this study are available on request.
