# Supplementary figures and images for: Camptocormia as a Phenotypic Variant of FSHD in the Elderly: Clinical, Genetic, and Imaging Features
Source: Eur J Neurol. 2025 Oct 2;32(10):e70332. doi: 10.1111/ene.70332 (PMC12490655; doi:10.1111/ene.70332)

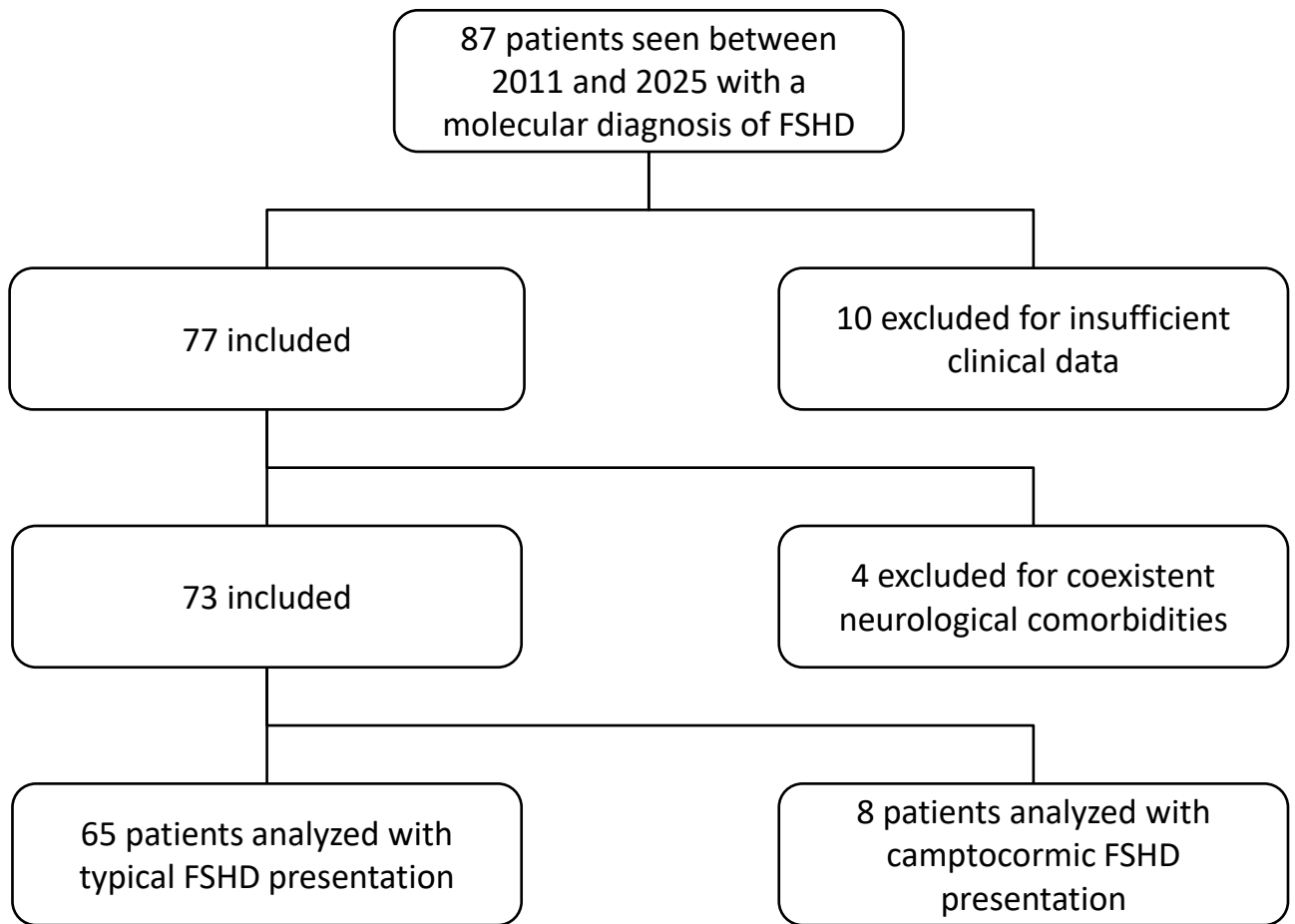

**Supplementary Figure 1.** Flow-chart of the study.

Supplement: Supplementary file 1 — Figure S1: Flowchart of the study. [file ENE-32-e70332-s002.pdf]
