# Supplementary material for: Camptocormia as a Phenotypic Variant of FSHD in the Elderly: Clinical, Genetic, and Imaging Features
Source: Eur J Neurol. 2025 Oct 2;32(10):e70332. doi: 10.1111/ene.70332 (PMC12490655; doi:10.1111/ene.70332)
Supplement: Supplementary file 2 — Figure S2: Spectrum of muscle imaging abnormalities in FSHD camptocormia. [file ENE-32-e70332-s003.pdf]

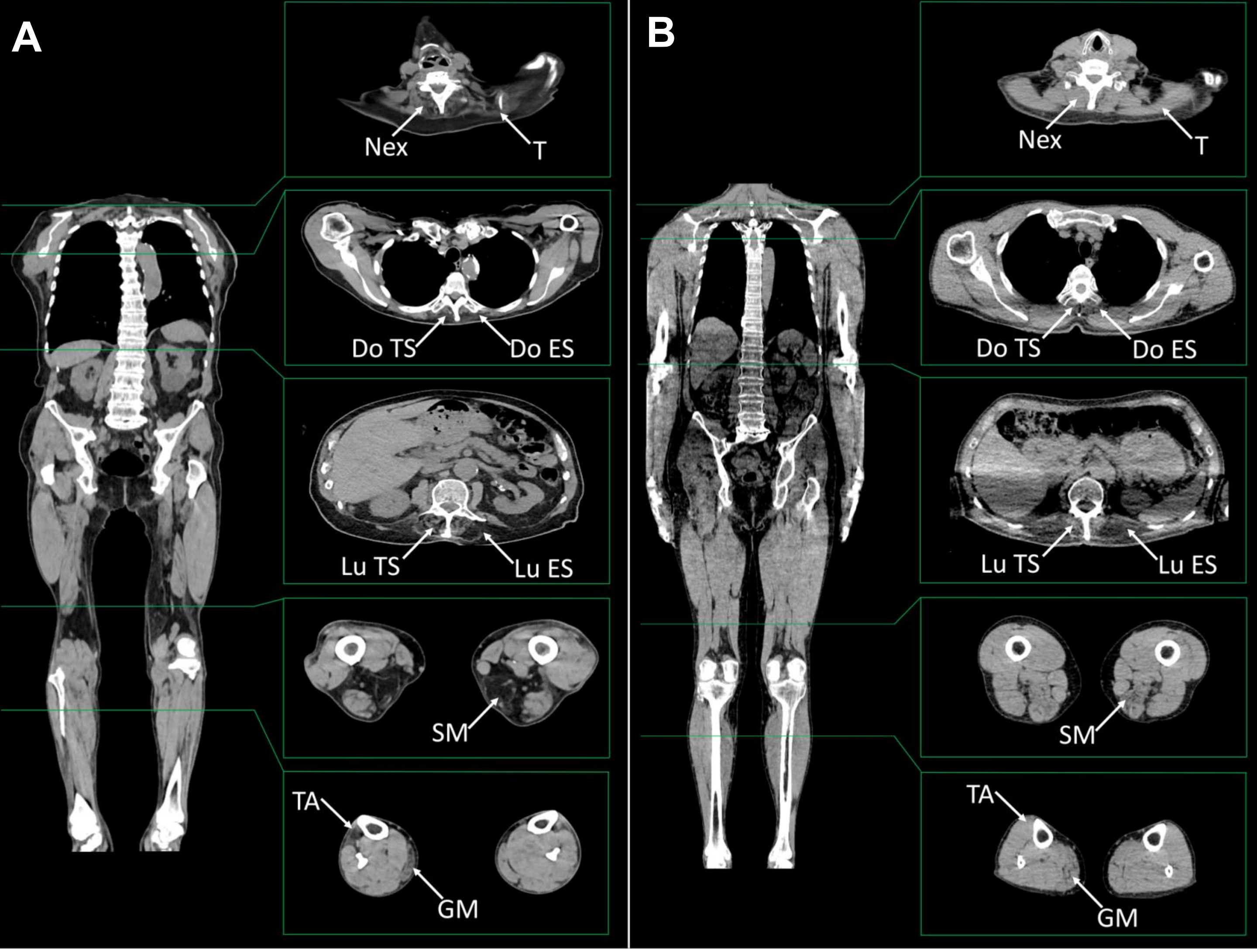

Supplementary Figure 2. Spectrum of muscle imaging abnormalities in FSHD camptocormia. Muscle CT scans of two patients with FSHD camptocormia. Axial slices are shown at different levels, from cranial (top) to caudal (bottom). (**A**) Case 1 showing severe involvement of muscles commonly affected by typical FSHD (T: trapezius, SM:semimembranosus, TA: tibialis anterior, GM: gastrocnemius medialis) in addition to the axial muscles typically involved in FSHD camptocormia (Nex:neck extensors; Do TS and Lu TS:dorsal and lumbar transversospinalis; Do and Lu ES: dorsal and lumbar erector spinae). (**B**) Case 2 presenting a selective involvement of axial muscles, with relative sparing of other muscle groups commonly affected in typical FSHD, including the trapezius. In both patients fatty replacement of the erector spinae muscles is more severe compared to the transversospinalis muscles.
