# Supplementary material for: Camptocormia as a Phenotypic Variant of FSHD in the Elderly: Clinical, Genetic, and Imaging Features
Source: Eur J Neurol. 2025 Oct 2;32(10):e70332. doi: 10.1111/ene.70332 (PMC12490655; doi:10.1111/ene.70332)
Supplement: Supplementary file 3 — Table S1: FSHD clinical scores and clinical categories of FSHD camptocormic patients. [file ENE-32-e70332-s001.docx]

| **PATIENT** | **FSHD CLINICAL SCORE** | **CLINICAL CATEGORY** |
| --- | --- | --- |
| 1 | 6 | D1 |
| 2 | 0 | D2 |
| 3 | 3 | D1 |
| 4 | 4 | D2 |
| 5 | 2 | D2 |
| 6 | 2 | D1 |
| 7 | 8 | D1 |
| 8* | 6 | D1 |

**Supplementary table.** FSHD clinical scores and clinical categories of FSHD camptocormic patients.

* Beevor sign assessment was not available for this patient, for which the FSHD clinical score has been calculated without this information.
